# Assessment of Inflammatory and Oxidative Stress Biomarkers for Predicting of Patients with Asymptomatic Carotid Artery Stenosis

**DOI:** 10.3390/jcm14030755

**Published:** 2025-01-24

**Authors:** Abdullah Burak Karaduman, Sinem Ilgın, Özlem Aykaç, Mehmetcan Yeşilkaya, Serkan Levent, Atilla Özcan Özdemir, Gozde Girgin

**Affiliations:** 1 Department of Pharmaceutical Toxicology, Faculty of Pharmacy, Anadolu University, Eskisehir 26470, Turkey; abkaraduman@anadolu.edu.tr; 2 Department of Pharmaceutical Toxicology, Faculty of Pharmacy, Hacettepe University, Ankara 06800, Turkey; ggirgin@hacettepe.edu.tr; 3 Department of Neurology, Faculty of Medicine, Eskisehir Osmangazi University, Eskisehir 26040, Turkey; oaykac@ogu.edu.tr (Ö.A.); mehmetcan.yesilkaya@ogu.edu.tr (M.Y.); ozcanozdemir@ogu.edu.tr (A.Ö.Ö.); 4 Department of Analytical Chemistry, Faculty of Pharmacy, Anadolu University, Eskisehir 26470, Turkey; serkanlevent@anadolu.edu.tr

**Keywords:** asymptomatic carotid artery stenosis, inflammatory biomarkers, oxidative stress biomarkers, tryptophan/kynurenine pathway, neopterin

## Abstract

**Background/Objectives**: Asymptomatic carotid artery stenosis is usually detected by physicians in patients, coincidentally, during an ultrasound examination of the neck. Therefore, measurable biomarkers in blood are needed to define the presence and severity of atherosclerotic plaque in patients to identify and manage it. We hypothesized that biomarkers that indicate pathways related to the pathogenesis of atherosclerosis could be used to identify the presence and severity of atherosclerotic plaque. For this purpose, the levels of participants’ inflammatory and oxidative stress biomarkers were determined. Kynurenine/tryptophan and neopterin levels were measured as relatively new biomarkers of inflammation in this study. **Methods**: Our study included 57 patients diagnosed with asymptomatic carotid artery stenosis and 28 healthy volunteers. Blood kynurenine and tryptophan levels were measured with LCMS/MS. Blood catalase, total superoxide dismutase (t-SOD), glutathione peroxidase (GPx), malondialdehyde, and neopterin levels were measured using the ELISA assay method. **Result**: The kynurenine/tryptophan ratio reflecting IDO activity was higher in patients than in healthy volunteers. Decreased tryptophan levels and increased kynurenine and neopterin levels were observed in patients who underwent carotid endarterectomy. In patients, catalase, t-SOD, and malondialdehyde levels were higher, while GPx activity was lower. These differences were found to be more significant in patients who underwent carotid endarterectomy. **Conclusions**: Increased kynurenine/tryptophan ratio and neopterin levels in patients with asymptomatic carotid artery stenosis were associated with the inflammatory status of the patients. Oxidative stress and inflammatory biomarkers can be considered effective diagnostic and severity indicators for asymptomatic carotid artery stenosis.

## 1. Introduction

Asymptomatic carotid artery stenosis is frequently characterized by atherosclerotic constriction of the carotid artery without a history of ischemic stroke, transient ischemic attack, or other neurologic symptoms related to the carotid arteries [1,2,3]. Carotid artery stenosis is responsible for around 10–20% of all ischemic strokes, the leading cause of death and disability worldwide [4,5]. This occurs through two primary mechanisms: hemodynamic impairment in cases of considerable stenosis and thromboembolism from an atherosclerotic plaque, regardless of the extent of stenosis. The degree of stenosis is a relevant risk factor for ischemic stroke [4]. Narrowing between 50% and 69% is classified as moderate stenosis, while narrowing exceeding 70% is typically classified as severe [6]. Certainly, carotid artery stenosis primarily focuses on avoiding strokes; it is important to identify and treat the degree of carotid artery stenosis to avert complications [7]. Doppler ultrasound is one of the standard diagnostic tools for carotid artery stenosis. However, a robust and clinically validated biomarker in the blood that may accurately diagnose patients and predict unfavorable outcomes in these individuals has not yet been identified [8]. As known, atherosclerosis, a chronic and local immune system response, constitutes the underlying pathology of carotid artery stenosis [9,10]. The most promising biomarkers are the ones that closely correlate with the pathophysiological process of the disease. In this context, focusing on the pathways involved in the initiation and progression of atherosclerosis may enable the identification of new diagnostic and prognostic biomarkers.

Given the significant role inflammation plays in every stage of the atherosclerotic process, atherosclerosis is typically regarded as a chronic inflammatory disease [11,12,13]. Flow-mediated inflammatory alterations in endothelial cells are believed to accompany the atherogenic process [14,15]. Adhesion molecules, chemoattractants, and other inflammatory factors are expressed by activated endothelial cells [14]. These factors attract lymphocytes and monocytes that adhere to the endothelium and infiltrate the arterial wall, causing inflammation [14,16]. This process involves a variety of cells and cytokines, including adhesion molecules, tumor necrosis factor (TNF-α), vascular smooth muscle cells, dendritic cells, endothelial cells, macrophages, lymphocytes (T and B cells), and interleukins. Pro-inflammatory cytokines, such as IL-1β, IL-6, TNF-α, and IFN-γ, are crucial in the inflammation associated with atherosclerosis [14,17]. Numerous inflammation-associated biomarkers, especially TNF-α and interleukin-6, have been identified as inflammatory biomarkers for monitoring atherosclerosis and cardiovascular risk [18,19]. In addition to cytokines, prostaglandins [20,21] and leukotrienes [22,23,24] have been investigated as therapeutic targets and inflammatory biomarkers in the development and progression of atherosclerosis. Also, kynurenines and neopterin are two of these research topics, mainly due to their role in inflammatory processes. The kynurenines, metabolites of the kynurenine pathway, which is the main pathway of metabolism of essential amino acid tryptophan, have been shown to have immune-modulatory properties and to be associated with inflammatory diseases [25,26,27,28]. The pro-inflammatory cytokine IFN-γ, associated with immune responses at all stages of atherosclerosis, regulates the kynurenine pathway in macrophage and dendric cells [25,29,30,31]. But, while an increasing number of studies indicate alterations in the kynurenine pathway in cardiovascular diseases [31,32,33,34,35,36,37] the precise role of kynurenines in their initiation and progression remains unclear. Also, neopterin, which is a pteridine derived from guanosine triphosphate, is produced by macrophages and dendritic cells by IFN-γ stimuli [38,39,40]. Studies have shown that plasma neopterin levels increased in patients with coronary artery disease [41,42,43,44], unstable angina [45], and stable angina pectoris [42,46,47]. Extensive expression of neopterin within atherosclerotic lesions in the arteries was evidenced [47,48]. On the other hand, neutrophil-to-lymphocyte ratio and red blood cell distribution width should also be emphasized as valuable markers of the systemic inflammatory response [49,50]. Several studies have demonstrated elevated levels of neutrophil-to-lymphocyte ratio [49,50,51,52] and red blood cell distribution width [53,54,55,56] in individuals with carotid atherosclerotic plaques, underscoring their significance in this context. Finally, some studies have demonstrated that circulating microRNAs (miRNAs) are effective diagnostic tools for detecting subclinical carotid atherosclerosis [57,58,59]. These miRNAs regulate various biological processes, including inflammation, endothelial function, and lipid metabolism, which are critical in the development of atherosclerosis. Their presence and altered expression levels in the bloodstream make them promising non-invasive diagnostic tools for early detection and risk assessment [60,61].

As known, atherosclerosis begins with the accumulation of lipoproteins, especially LDL, in the arterial wall, with subsequent LDL oxidation into oxidized LDLs [29,62,63]. Besides the importance of this process, the imbalance between oxidants and antioxidants is also important for the progression of atherogenesis [64]. The overproduction of reactive oxygen species results in oxidative stress, a significant contributing factor to the development and progression of atherosclerosis. Reactive oxygen species are crucial in preserving vascular health due to their potent signaling abilities. Nevertheless, reactive oxygen species also trigger pro-atherogenic mechanisms such as inflammation, endothelial dysfunction, and disrupted lipid metabolism. Additionally, the failure or overwhelming of endogenous antioxidant systems is a common feature in atherosclerosis that promotes oxidative stress [65].

The release of inflammatory chemokines and cytokines that lead to the overactivation of the inflammatory response and the overgeneration of reactive oxygen species that lead to a state of oxidative stress are highlighted as major risk factors for the development and severity of atherosclerosis. Therefore, we focused on alterations in inflammatory and oxidative stress biomarker levels in patients with asymptomatic carotid artery stenosis compared to healthy volunteers. Additionally, it is particularly noteworthy that few studies describe changes in tryptophan/kynurenine and neopterin levels in carotid artery stenosis. Therefore, kynurenine/tryptophan and neopterin levels were measured as relatively new inflammatory biomarkers to determine their diagnostic and severity of asymptomatic carotid artery stenosis.

## 2. Materials and Methods

### 2.1. Study Groups

This prospective, cross-sectional study was conducted at the Neurology Outpatient Clinic of Eskisehir Osmangazi University Hospital from December 2021 to November 2023. One hundred and two people were admitted to the neurology outpatient clinic during this period. While carotid artery stenosis was not detected in 35 people, 67 people were diagnosed with asymptomatic carotid artery stenosis by vascular neurologists. Using a standardized questionnaire, we obtained information about demographic variables, previous disease history, and smoking through face-to-face clinic interviews. Healthy volunteers and patients with asymptomatic carotid artery stenosis aged 40–80 were included. People who have been diagnosed with an inherited metabolic disease, who have undergone major surgery or trauma within the previous 3 months, who have chronic inflammatory disease, congestive heart failure, liver failure, hematological disorders, kidney failure, or any history of malignancy were excluded from the study. It was determined that seven healthy volunteers and ten patients had at least one of the diseases mentioned; therefore, these people were excluded from the study. Finally, our study included 57 patients with asymptomatic carotid artery stenosis and 28 healthy volunteers. It should be noted that power analysis for each parameter evaluated in the study was performed using SPSS v.23 (one-way ANOVA), and the power for all parameters exceeded 80% at a 5% error level. Smoking was defined as smoking more than 3 cigarettes per day for more than six months. Hypertension and diabetes were defined as the subject having been previously diagnosed with the disease in the hospital or taking the medication regularly. Obesity status was determined using body mass index (BMI). BMI was calculated as weight (kg) divided by height squared (m^2^). Individuals with a BMI over 30 kg/m^2^ were classified as obese.

Carotid artery stenosis was diagnosed through Doppler ultrasonography and Computed Tomography (CT) angiography performed by vascular neurologists in the specialized stroke unit at the hospital. Neurologists with at least 5 years of experience in vascular and interventional neurology meticulously determine patients’ treatment protocols according to current guidelines, taking into account factors such as comorbidities, stenosis levels, stenotic plaque characteristics, and medication history [66]. In our study, carotid artery stenosis was defined primarily based on the degree of luminal narrowing, with a minimum threshold of 50% stenosis considered clinically significant. This determination was made using duplex ultrasound, which quantifies the percentage reduction in vessel diameter relative to a healthy reference segment. Beyond the degree of stenosis, we also incorporated high-risk plaque features—such as ulceration, intraplaque hemorrhage, and echo-lucency—into our evaluation, given their strong association with an increased risk of cerebrovascular events. Additionally, hemodynamic parameters, including elevated peak systolic velocity and the detection of microemboli using transcranial Doppler, were utilized to provide further insights into the clinical relevance of the stenosis. By combining anatomical measurements with hemodynamic findings, our approach aimed to deliver a comprehensive and nuanced assessment of carotid stenosis to guide diagnostic and therapeutic decision-making effectively. Also, several studies have employed approaches similar to ours in assessing asymptomatic carotid artery stenosis by integrating anatomical measurements, hemodynamic findings, and plaque characteristics to inform treatment decisions [2,67]. In our study, all patients with asymptomatic carotid artery stenosis were divided into three groups according to treatment protocols to determine changes in biomarker levels according to the severity of atherosclerosis. In our study, all patients with asymptomatic carotid artery stenosis were divided into three groups based on their treatment protocols to determine changes in biomarker levels according to the severity of atherosclerosis. The first group was patients with medical follow-up by drug treatment (**Group 1**; n:27). The second group was patients who underwent stenting (**Group 2**; n:18). The third group was patients who underwent carotid endarterectomy (**Group 3**; n:12).

### 2.2. Ethical Approval

The research protocol received ethical approval from the Eskişehir Osmangazi University Non-Interventional Clinical Research Ethics Committee (No. 2021/12, No. 08, 14 December 2021). The Helsinki Declaration conducted the study. Participants provided informed consent before their involvement in the study.

### 2.3. Biochemical Parameters

Blood samples were collected from patients before medical treatment and carotid artery interventional management. Bloods were collected into tubes with EDTA and centrifuged at 2000× *g* for 10 min at 4 °C. Prepared plasma samples were separated into vials in portions and stored at −80 °C until analyzed.

Blood samples for total cholesterol, triglyceride, low-density lipoprotein (LDL), high-density lipoprotein (HDL), and C-reactive protein (CRP) were taken in the morning and analyzed using standardized procedures at the hospital’s central biochemistry laboratory. Tryptophan and kynurenine levels were measured using LC-MS/MS (Shimadzu Corporation, Kyoto, Japan). Indoleamine 2,3-dioxygenase (IDO) enzyme activity was calculated using plasma kynurenine and tryptophan levels and presented as µmol kynurenine to µmol tryptophan.

Neopterin, catalase, total superoxide dismutase, glutathione peroxidase, and malondialdehyde levels were determined using commercially available human enzyme-linked immunosorbent assay (ELISA) kits. The neopterin ELISA kit was purchased from the Bioassay Technology Laboratory (Shanghai, China). In contrast, the others were purchased from the Elabscience Biotechnology Inc. (Houston, TX, USA). All ELISAs were performed according to the manufacturer’s instructions with commercially available kits.

### 2.4. Tryptophan and Kynurenine Analysis

Trichloroacetic acid, formic acid, acetonitrile, and potassium phosphate dibasic trihydrate were purchased from Merck KGaA (Darmstadt, Germany). L-tryptophan and L-kynurenine were purchased from the Cayman Chemical Company (Ann Arbor, MI, USA). All reagents utilized in the experiment were of analytical grade or higher purity, and water of HPLC grade was used to prepare all aqueous solutions.

LCMS/MS analysis was performed using an LCMS-8040 system. Tryptophan calibration standards were prepared in the 10–100 μmol/L range by sequential dilution of solutions. Kynurenine calibration standards were prepared in the 0.4–8 μmol/L range by sequential dilution of solutions. A total 400 µL plasma sample was diluted with 400 µL phosphate buffer. The samples were deproteinized by adding 100 µL 2 M trichloroacetic acid solution. After vortexing, the samples were centrifuged at 13,000 rpm. The supernatants were transferred into vials and injected into the LC-MS/MS system. A mixture of water containing 0.1% formic acid and acetonitrile containing 0.1% formic acid (65/35, *v*/*v*) was used as the mobile phase. The total injection volume was 0.3 µL, and the flow rate was 0.3 mL/min. Chromatographic separation was performed using an isocratic gradient with Nucleosil HPLC Column (Teknokroma Brisa LC2 C18 Column 3 μm 15 × 0.46 cm, Teknokroma Analytica S.A., Barcelona, Spain) maintained at 40 °C. The multiple-reaction monitoring (MRM) method in positive mode for LCMS analysis was performed, and the parameters are given in Table 1. Additionally, sample chromatograms and MRM spectrums of standards conducted by the developed method were given as supplementary data.

### 2.5. Statistical Analysis

IBM SPSS Statistics v. 23 (IBM Corporation—International Business Machines Corporation of Armonk, New York, NY, USA) was utilized for statistical analysis. The Shapiro–Wilk test was employed to assess the normal distribution of the variables. Demographic data were analyzed using Student’s *t*-test, one-way ANOVA test, or Pearson’s chi-squared test and were presented as numbers or mean ± standard deviation. Other parameters were analyzed using Student’s *t*-test or one-way ANOVA test and were presented as mean ±standard deviation data. When the ANOVA test was statistically significant, the post hoc Tukey and LSD tests were performed. All tests were defined as statistically significant at a *p*-value less than 0.05. Post hoc power analysis was performed after the completion of the study to evaluate the adequacy of the sample size. The analysis was also conducted using SPSS software (version 23), assuming an alpha error probability of 0.05 and Cohen’s effect size of 0.4. The power of the study was calculated for all evaluated parameters and found to exceed 80%.

## 3. Results

### 3.1. Demographic Data and Characteristics of Participants

The demographic data and characteristics of the participants are presented in Table 2. Twenty-eight healthy volunteers and 57 patients with asymptomatic carotid artery stenosis were recruited in this study. The healthy volunteers and patients did not differ in terms of gender. However, the patients’ median age was significantly higher than the healthy volunteers. No statistical difference was found between the groups in terms of comorbid diseases such as hypertension, diabetes, and obesity.

### 3.2. Lipid Profiles of Participants

The levels of triglycerides and HDL did not differ significantly between healthy volunteers and patients with asymptomatic carotid artery stenosis. However, compared to the healthy volunteers, a statistically significant reduction in total cholesterol and LDL levels was observed in patients. No statistically significant differences were observed in terms of triglyceride, LDL, and HDL levels in patients who underwent medical follow-up, stenting, and endarterectomy compared to healthy volunteers. However, a statistically significant decrease was observed in terms of cholesterol levels in patients who underwent medical follow-up, stenting, and endarterectomy compared to healthy volunteers (Table 3). The potential cause for the reduced cholesterol levels observed in patients could be the pharmaceutical interventions employed by the patients to manage their dyslipidemia. In fact, during in-person interviews, it was determined that patients diagnosed with carotid artery stenosis were using at least one antihyperlipidemic drug.

### 3.3. Inflammatory Biomarkers of Participants

When CRP, neopterin, and kynurenine levels were compared to healthy volunteers, increased CRP, neopterin, and kynurenine levels were observed in patients with asymptomatic carotid artery stenosis, but these differences were not considered statistically significant. Tryptophan levels were statistically significantly decreased in patients with asymptomatic carotid artery stenosis compared to the healthy volunteers. Statistically significant increases in the kynurenine/tryptophan ratio that reflects IDO activity were noted in the patients with asymptomatic carotid artery stenosis compared to the healthy volunteers. No statistically significant difference was observed in terms of CRP levels in patients who underwent medical follow-up, stenting, and endarterectomy compared to healthy volunteers. Neopterin and kynurenine levels were statistically significantly increased in the patients who underwent carotid endarterectomy compared with healthy volunteers. Additionally, neopterin and kynurenine levels were statistically significantly higher in the patients who underwent carotid endarterectomy than in other patients. Tryptophan levels were statistically significantly decreased in patients who underwent medical follow-up, stenting, and endarterectomy compared to the healthy volunteers. Statistically significant increases in IDO activity were noted in patients who underwent stenting and endarterectomy compared to the healthy volunteers. Additionally, IDO activity was statistically significantly higher in the patients who underwent carotid endarterectomy than in other patients (Table 4).

### 3.4. Oxidative Stress Biomarkers of Participants

When malondialdehyde levels were compared to healthy volunteers, increased malondialdehyde levels were observed in patients with asymptomatic carotid artery stenosis, but these differences were not considered statistically significant. Catalase and superoxide dismutase activities were statistically significantly increased in patients with asymptomatic carotid artery stenosis compared to the healthy volunteers. However, glutathione peroxidase activities were statistically significantly decreased in the patients with asymptomatic carotid artery stenosis compared to the healthy volunteers. Statistically significant increases in catalase activities were noted in patients who underwent medical follow-up, stenting, and endarterectomy compared to the healthy volunteers. Additionally, catalase activity was statistically significantly higher in the patients who underwent carotid endarterectomy than in other patients. Increases in total superoxide dismutase activity were statistically significant in patients who underwent medical follow-up and carotid endarterectomy. Statistically significant decreases in glutathione peroxidase activity were noted in patients who underwent medical follow-up and endarterectomy compared to the healthy volunteers. Malondialdehyde levels were statistically significantly increased in the patients who underwent carotid endarterectomy compared to the healthy volunteers (Table 5).

## 4. Discussion

Atherosclerosis is a chronic inflammatory condition that primarily affects the walls of large and medium arteries, including the carotid arteries [68]. Many factors contribute to the pathogenesis of atherosclerosis, the most important of which is inflammation [69]. Inflammation plays a significant role in all stages of atherosclerosis [70,71]. Therefore, several biomarkers linked to vascular inflammation have been discovered as new targets for monitoring atherosclerosis. The CRP is a well-researched and widely accepted biomarker that indicates inflammation [18,72]. The CRP has proven to be a predictive marker for cardiovascular events, independently and in combination with other parameters [73]. Also, many studies have examined the relationship between CRP levels and carotid artery stenosis. These studies found increased CRP levels associated with carotid artery stenosis [74,75,76,77]. In our study, although plasma CRP levels increased in patients with asymptomatic carotid artery stenosis, this increase was not considered statistically significant. At this point, it should be emphasized that increased CRP levels may also triggered by many disorders unrelated to cardiovascular disease, which interferes with clinical application [18,72]. So, while CRP has been extensively studied as a marker of inflammation and cardiovascular risk, its specificity for atherosclerosis has been questioned. To enhance risk assessment and management in atherosclerotic disease, there is a growing consensus in the scientific community for developing and validating new biomarkers that can provide more targeted and accurate information on the presence and severity of atherosclerosis.

At this point, it should be emphasized that cytokines can affect every stage of atherosclerosis development, from the early recruitment of circulating monocytes and other immune cells from the bloodstream to the formation and stability of mature plaque [29,63,78]. Cytokines can be produced by almost all cell types in this context, particularly helper T cells and macrophages [79]. During inflammation, pro-inflammatory cytokines, primary IFN-γ, increase the catabolism of tryptophan by encouraging the expression of IDO in macrophages and other cells [26,27,28,80,81,82]. So, under inflammatory conditions, IDO activation leads to an increase in tryptophan metabolism to kynurenine [28,83]. It has been reported that the kynurenine pathway, which is the main pathway responsible for the metabolism of the essential amino acid tryptophan, plays a crucial role in regulating vascular inflammation [25,29,30,84]. Also, a relationship between IDO activity and the development of atherosclerosis has been demonstrated [28,82,85]. For biomonitoring of the metabolism of tryptophan to kynurenine, the ratio of blood kynurenine concentration to blood tryptophan concentration is used, and this ratio reflects IDO activity [31,80,86,87]. In studies, biomarkers defining the tryptophan–kynurenine pathway especially increased the kynurenine/tryptophan ratio, so IDO activity is associated with the presence of atherosclerotic plaques [28,83,88]. Our study determined that IDO activity, reflected by the plasma kynurenine/tryptophan ratio, was increased in patients with asymptomatic carotid artery stenosis compared to healthy volunteers. Additionally, more IDO activities were observed in patients who underwent carotid endarterectomy compared to others. Therefore, IDO activity can be considered a biomarker that can be used to determine both the presence and severity of atherosclerosis. Studies have determined that increased carotid artery intima/media thickness is accompanied by an increased kynurenine/tryptophan ratio [89,90]. On the other hand, studies have observed that IDO activity is increased in patients with cardiovascular diseases compared to healthy volunteers [91,92,93,94,95]. Prospective study results have been interpreted as kynurenine pathway metabolites may be useful in predicting the onset and progression of atherosclerotic pathologies. The results have pointed out that tryptophan levels tend to decrease, and kynurenine levels tend to increase in disease states [31,88,96,97,98,99].

Neopterin is biosynthetically derived from guanosine triphosphate by guanosine triphosphate cyclohydrolase 1. In macrophages, cyclohydrolase I activity is upregulated mainly by IFN-γ [100,101]. Neopterin levels significantly increase in any condition involving immune response activation and inflammation; therefore, it is accepted as a biomarker for activating monocytes/macrophages [100,102,103]. Under inflammatory conditions, IFN-γ increases neopterin synthesis by inducing the expression of guanosine triphosphate cyclohydrolase 1 [104,105]. Our study measured neopterin levels higher in patients with asymptomatic carotid artery stenosis than in healthy volunteers. Additionally, more neopterin levels were noted in the patients who underwent carotid endarterectomy than in other patients. Therefore, it should be said that neopterin level may indicate both the presence and severity of stenosis. Other studies have also shown increased circulating neopterin levels in patients with carotid artery stenosis [106,107,108]. Furthermore, other research findings have also indicated a possible correlation between neopterin levels and the degree of carotid artery stenosis in patients [47,109,110,111].

Oxidative stress is a widely recognized factor in the development of atherosclerosis, which co-occurs with the activation of pro-inflammatory signaling pathways [112]. Oxidative modification of LDL accumulated in the artery wall as a critical process of atherosclerosis initiates atherosclerosis development, promoting the vascular inflammatory response [65,113,114]. Regarding vascular inflammatory response, migrating immune cells to the arterial wall produces further reactive oxygen species. Therefore, vascular inflammation and oxidative stress cannot separate each other in the development and progression of atherosclerotic plaque [112]. As mentioned above, currently, there is no agreement on the definition of an optimal biomarker that can predict the risk of stroke in patients with asymptomatic carotid artery stenosis. Therefore, targeting biomarkers of oxidative stress as a causal factor of atherosclerosis appears to be a plausible approach. Oxidative stress can be monitored using different biomarkers, such as malondialdehyde, one of the lipid oxidation products. Also, previous research has established a correlation between atherosclerosis and an elevation in malondialdehyde levels [113,115,116]. Our study also drew attention to increased malondialdehyde levels in patients with asymptomatic carotid artery stenosis, especially patients who underwent carotid endarterectomy, compared to healthy volunteers. On the other hand, antioxidants are crucial in preventing oxidant formations, intercepting oxidants after they have formed, and repairing damage caused by oxidants [117]. Superoxide dismutase, glutathione peroxidases, and catalase, as some of the key enzymes of the antioxidant system, regulate the formation and degradation of reactive oxygen species in vascular cells [114]. Our results demonstrated lower glutathione peroxidase activity but higher superoxide dismutase and catalase activities in patients with asymptomatic carotid artery stenosis compared to healthy volunteers. Compared to healthy individuals, the altered activities of these enzymes in patients suggest a dysregulation in the antioxidant defense system, potentially contributing to the pathogenesis of carotid artery stenosis. At this point, it may be specified that homeostatic up-regulation of the antioxidant enzyme system in response to increased free radicals may occur to prevent vascular damage [118,119]. In summary, the disrupted oxidant–antioxidant system, specifically lower glutathione peroxidase and higher malondialdehyde, superoxide dismutase, and catalase activities, in patients with asymptomatic carotid artery stenosis compared to healthy volunteers highlight the potential role of oxidative stress in the development and progression of carotid artery stenosis. Further exploration of these enzyme systems and their implications in carotid artery stenosis could offer valuable insights for developing innovative diagnostic and therapeutic approaches for this condition. On the other hand, it was also observed that oxidative stress was induced more in the patients who underwent carotid endarterectomy than in other patients. Therefore, biomarkers of oxidative stress may also indicate the severity of stenosis. In addition, the balance between reactive oxygen species and antioxidant enzymes is disrupted in patients with asymptomatic carotid artery stenosis.

## 5. Conclusions

Our study has identified kynurenine, tryptophan, and neopterin as significant biomarkers for asymptomatic carotid artery stenosis, 10–20% of all ischemic strokes leading cause of death and disability worldwide. These results may provide new insight into developing a more effective diagnosis and treatment strategy to heal and/or prevent the progression of carotid artery stenosis. At this point, investigating the therapeutic capacity of immunomodulation in carotid artery stenosis may be considered a critical approach. On the other hand, the data mentioned above suggest that oxidative stress was elevated in patients, as evidenced by the notable alterations in the levels of oxidative stress markers. Our results need to be supported by extensive clinical studies. Studies with larger sample sizes are required to better define the significance of biomarkers in the diagnosis and severity of carotid artery stenosis. On the other hand, studies are also needed to determine the value of inflammatory and oxidative stress biomarkers in predicting the progression of asymptomatic carotid artery stenosis.

## 6. Limitations

The small sample size of our study, especially in subtypes of carotid artery stenosis, is the limitation. The applicability of biomarkers in indicating the severity of damage is uncertain due to the small sample size. At this point, it should be emphasized that it is quite difficult to reach large sample sizes because it is not possible to detect most of the patients before symptoms start. Studies with larger sample sizes are needed to better define the significance of biomarkers in the diagnosis and severity of carotid artery stenosis. On the other hand, the patients included in the study could not be followed up. Therefore, we do not know the value of the biomarkers measured in the study in predicting the prognosis of asymptomatic carotid artery stenosis. Hence, studies are also needed to define the value of inflammatory and oxidative stress biomarkers in predicting the progression of asymptomatic carotid artery stenosis. Another limitation of the study is that a power analysis was not conducted before the study began. The study sample size was determined considering the available resources and time constraints. However, the post hoc power analysis performed at the end of the study showed that the power of all the parameters examined was over 80%. However, the post hoc power analysis is only an indicator for evaluating the adequacy of the study based on the available data and may contain deficiencies in the sample size determinations at the planning stage.

## Figures and Tables

**Table 1:** MRM data for tryptophan and kynurenine.

	Precursor Ion	Daughter Ion	Dwell Time ms	Q1 Pre-Bias (V)	CE	Q3 Pre-Bias (V)
L-Tryptophan	205.10	188.05	100.0	−15.0	−12.0	−19.0
	205.10	146.05	100.0	−15.0	−22.0	−27.0
	205.10	117.90	100.0	−10.0	−27.0	−25.0
L-Kynurenine	209.10	192.15	100.0	−10.0	−8.0	−12.0
	209.10	146.05	100.0	−23.0	−22.0	−27.0
	209.10	94.05	100.0	−14.0	−17.0	−18.0

Precursor ion: An ion that is the source of fragmentation, either spontaneous or induced by collisions. Daughter ion: An ion that is the charged product of an ion dissociation. Dwell time: Time that is the time spent acquiring the targeted MRM transition during each cycle. Q1 Pre-Bias: Voltage that promotes the ionization of the precursor ion. CE: The collision energy of the ions in the collision cell. Q3 Pre-Bias: Voltage which promotes the ionization of the product ion.

**Table 2:** Demographic data and characteristics of the participants.

	Gender (n)		Age (Mean ± SD)	Smoking (n)		Comorbidly (n)		
	Female	Male		Yes	No	Hypertensin	Diabetes	Obesity
**H**	18	10	60 ± 10	5	23	15	4	9
**ACAS**	21	36	67 ± 8 (*)	18	39	32	19	10
**Group 1**	12	15	66 ± 8 (*)	10	17	18	6	8
**Group 2**	6	12	65 ± 8 (*)	6	12	7	7	2
**Group 3**	3	9	74 ± 8 (*)	2	10	7	6	0

**H**, healthy volunteers; **ACAS**, patients with asymptomatic carotid artery stenosis; **Group 1**, patients with medical follow-up by drug treatment; **Group 2**, patients with stenting; **Group 3**, patients with carotid endarterectomy. Differences between groups were assessed using Student’s *t*-test, one-way ANOVA test, or Pearson’s chi-squared test. Values were presented as numbers or mean ± standard deviation. * Different from H (*p* < 0.05).

**Table 3:** Lipid profiles of participants.

	Total Cholesterol (mg dL^−^)	Triglyceride (mg dL^−^)	LDL (mg dL^−^)	HDL (mg dL^−^)
**H**	222.35 ± 39.51	168.24 ± 76	146.53 ± 36.41	44.00 ± 6.98
**ACAS**	174.30 ± 43.51 (***)	135.30 ± 54.27	118.58 ± 40.01 (*)	44.12 ± 10.73
**Group 1**	183.48 ± 42.98 (*)	126.62 ± 41.59	119.05 ± 35.86	46.43 ± 9.03
**Group 2**	173.11 ± 50.54 (*)	141.11 ± 58.16	114.58 ± 44.17	41.33 ± 12.26
**Group 3**	153.22 ± 18.40 (*)	142.36 ± 70.33	124.58 ± 45.38	43.80 ± 11.27

**H**, healthy volunteers; **ACAS**, patients with asymptomatic carotid artery stenosis; **Group 1**, patients with medical follow-up by drug treatment; **Group 2**, patients with stenting; **Group 3**, patients with carotid endarterectomy. Differences between groups were assessed using Student’s *t*-test or one-way ANOVA test. Values were presented as mean ± standard deviation. Abbreviations: LDL, Low-density lipoprotein; HDL, High-density lipoprotein. * Different from H (*p* < 0.05). *** Different from H (*p* < 0.001).

**Table 4:** Inflammatory biomarkers of participants.

	CRP (mg L^−^)	Neopterin (nmol L^−^)	Kynurenine (μmol L^−^)	Tryptophane (μmol L^−^)	IDO Activity
**H**	1.98 ± 0.89	3.18 ± 0.99	2.01 ± 0.22	45.38 ± 7.82	45.66 ± 4.68
**ACAS**	2.51 ± 1.49	3.33 ± 1.08	2.17 ± 0.43	35.58 ± 6.98 (***)	54.53 ± 9.99 (***)
**Group 1**	2.37 ± 0.96	3.23 ± 0.68	2.04 ± 0.22	37.17 ± 6.44 (*)	47.36 ± 5.48
**Group 2**	2.24 ± 1.38	2.85 ± 0.97	2.08 ± 0.44	36.40 ± 6.61 (*)	52.37 ± 5.27 (*)
**Group 3**	3.13 ± 2.28	4.28 ± 1.41 (*, +, !)	2.51 ± 0.59 (*, +, !)	30.07 ± 6.82 (*)	66.73 ± 7.76 (*, +, !)

**H**, healthy volunteers; **ACAS**, patients with asymptomatic carotid artery stenosis; **Group 1**, patients with medical follow-up by drug treatment; **Group 2**, patients with stenting; **Group 3**, patients with carotid endarterectomy. Differences between groups were assessed using Student’s *t*-test or one-way ANOVA test. Values were presented as mean ± standard deviation. Abbreviations: CRP, C-reactive protein; IDO, indoleamine 2,3-dioxygenase. * Different from H (*p* < 0.05). *** Different from H (*p* < 0.001). + Different from Group 1 (*p* < 0.05). ! Different from Group 2 (*p* < 0.05).

**Table 5:** Oxidative stress biomarkers of participants.

	CAT (U mL^−^)	T-SOD (U mL^−^)	GSH-Px (U mL^−^)	MDA (ng mL^−^)
**H**	36.66 ± 5.37	52.03 ± 10.45	1008.78 ± 188.38	80.75 ± 23.41
**ACAS**	244,052 ± 43.01 (***)	61.64 ± 10.68 (***)	634.91 ± 180.5 (***)	84.47 ± 33.93
**Group 1**	193.46 ± 74.88 (*)	61.49 ± 9.46 (*)	630.56 ± 230.18 (*)	82.40 ± 29.98
**Group 2**	177.28 ± 50.71 (*)	60.47 ± 13.40	584.94 ± 185.73 (*)	85.69 ± 39.30
**Group 3**	284.13 ± 74.7 (*, +, !)	63.78 ± 9.16 (*)	731.14 ± 202.83	90.66 ± 27.22 (*)

**H**, healthy volunteers; **ACAS**, patients with asymptomatic carotid artery stenosis; **Group 1**, patients with medical follow-up by drug treatment; **Group 2**, patients with stenting; **Group 3**, patients with carotid endarterectomy. Differences between groups were assessed using Student’s *t*-test or one-way ANOVA test. Values were presented as mean ± standard deviation. Abbreviations: CAT, catalase; T-SOD, total superoxide dismutase; MDA, malondialdehyde; GSH-Px, glutathione peroxidase. * Different from H (*p* < 0.05). *** Different from H (*p* < 0.001). + Different from Group 1 (*p* < 0.05). ! Different from Group 2 (*p* < 0.05).

## Data Availability

The data used in this study were initially collected in Excel format and subsequently converted to SPSS format for statistical analysis. If you wish to access the research data, please send an email to abkaraduman@anadolu.edu.tr, and the data will be provided upon request.
